# Supplementary material for: Estimating economic losses to tourism in Africa from the illegal killing of elephants
Source: Nat Commun. 2016 Nov 1;7:13379. doi: 10.1038/ncomms13379 (PMC5097124; doi:10.1038/ncomms13379)
Supplement: Supplementary Information — Supplementary Figures 1-3, Supplementary Table 1, Supplementary Notes 1-4 and Supplementary References [file ncomms13379-s1.pdf]

Supplementary Figures

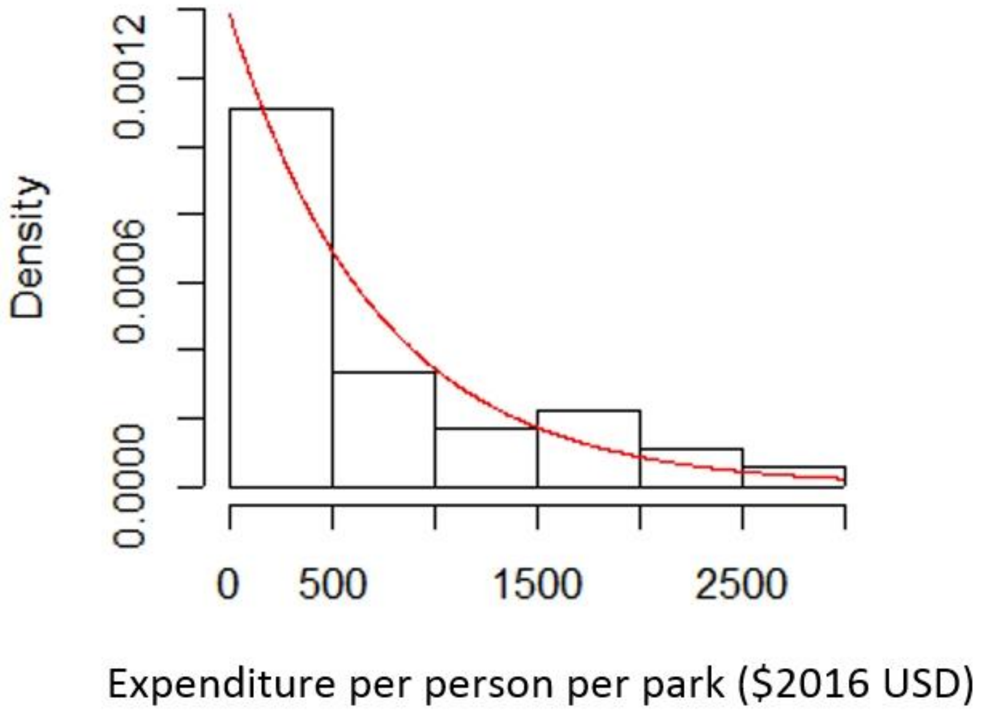

Supplementary Figure 1. The distribution of expenditures per-person per-park. Figures in $2016 USD (*n* = 36). The best fitting distribution, as assessed by the lowest AIC among 4 competing distributions was the exponential distribution, with rate parameter = 0.0014.

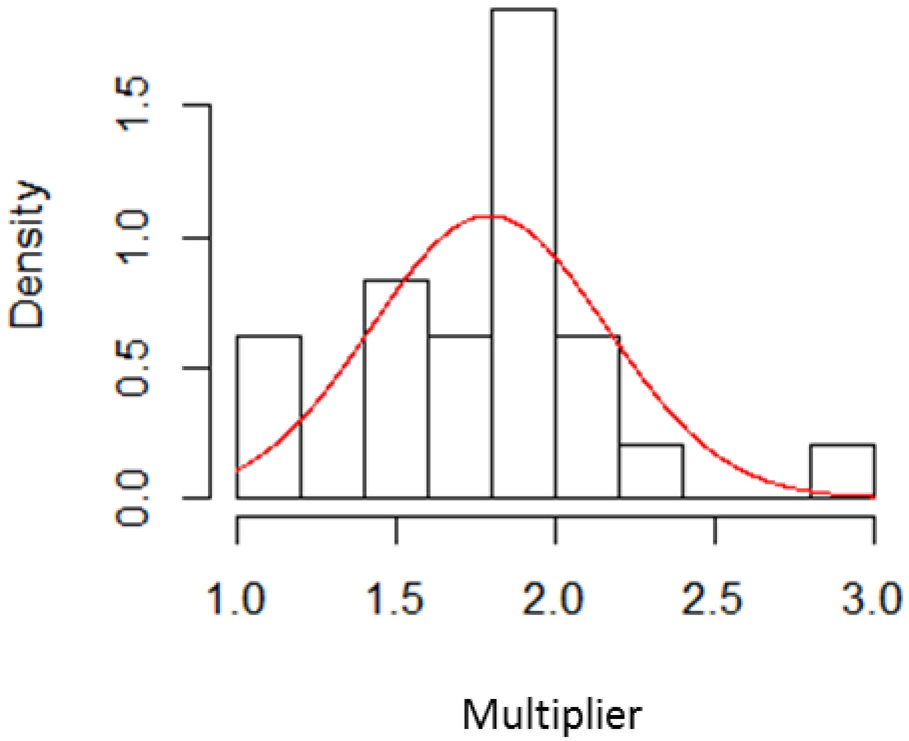

Supplementary Figure 2. The distribution of 'multiplier' effects for tourism in Africa. Values were taken from studies that estimated how tourist spending had indirect and induced local economic impacts (*n* = 24). The best fitting distribution, as assessed by the lowest AIC among 4 competing distributions was the normal distribution (mean = 1.793, standard deviation = 0.369).

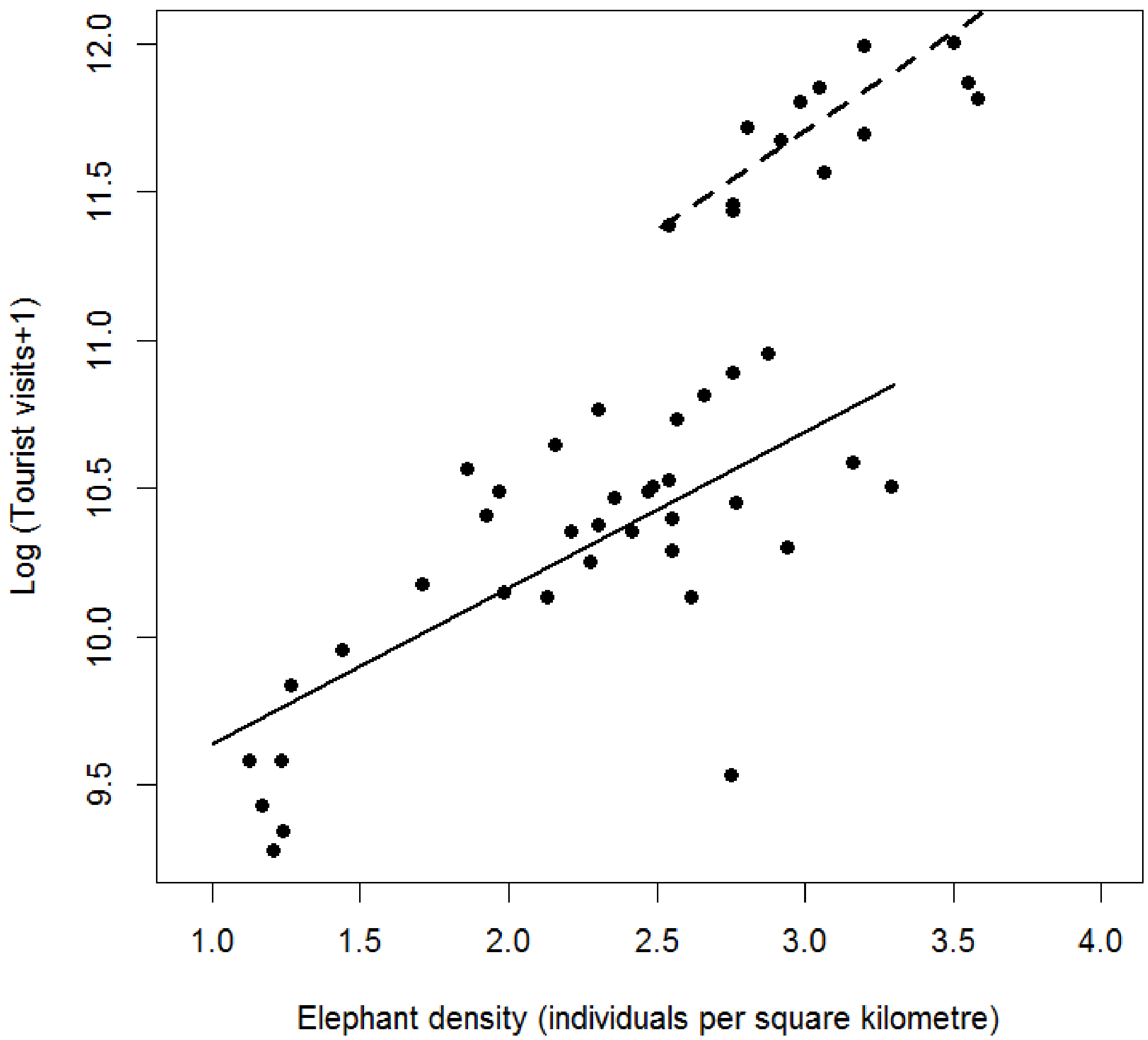

Supplementary Figure 3. Bivariate relationship between tourist visits (log-transformed) and elephant density at Addo Elephant National Park in South Africa. Lower line is data from 1956-1996 ($Y = 0.53*X + 9.11$, $R^2 = 0.50$, $P < 0.0001$), upper line 1997-2010 ($Y = 0.67*X + 9.71$, $R^2 = 0.68$, $P = 0.002$).

Supplementary Tables

Supplementary Table 1. Summary of variables used in Bayesian regression analysis.

Variable	Original Units	Log - transform?	Mean	Min	Max
Annual visits	# visitors per year	Y	7.01	0	14.20
Country PPP	USD 2006	Y	4.47	3.74	5.57
Elephant density	Individuals per km^2	N	0.47	0	5.23
Lion	1/0	N	0.48	0	1
Access	minutes to nearest city	Y	2.18	0	3.37
Forest	1/0	N	0.28	0	1
Surrounding population	# people within 100km buffer	Y	7.25	0	9.06
Area	km^2	Y	5.11	2.54	6.68
Attractiveness	1-5 scale	N	4.44	2.83	5.34

Dependent variable was annual visits, others are independent variables at African protected areas in elephant range state countries.

Supplementary Notes

Supplementary Note 1

Calculations for the value of ivory from poached elephants.

From 2010-2012, the most recent period for which data is available, estimates of annual poaching of elephants across Africa ranged from 21,000 – 41,000 individuals [1]. We use the central estimate of 33,630 poached elephants per year. The amount of ivory produced per elephant appears to be declining over time, so we use estimates of tusk weight for 2010-2012 that show an average weight of 6.23 kg per tusk from seizures during these years [2]. We also use an average of 1.88 tusks per elephant, as not all elephants carry two tusks [3]. Ivory prices fluctuate over time, so we averaged separate estimates for 2010 [4], 2011 [5], and 2012 [6] to arrive at an average value of $1514 per kg on the Chinese market for these three years. Combining these values results in an estimate that the annual value of raw ivory emanating from Africa was worth $596.8 million (2016 USD) during the 2010-2012 period.

Supplementary Note 2

Tourism and elephant densities at Addo Elephant National, South Africa.

Our Bayesian regression models of the relationship between tourist visits and elephant densities at Addo Elephant National Park included potentially confounding variables that changed over time. The results showed that after controlling for these additional independent variables, elephant densities were positively associated with tourist visits during the period 1954-2010, and also during the two distinct time phases apparent in Supplementary Fig. 3 (1956-1995 and 1996-2010). Note that our results contradict those of the original study from which we drew the data [6], in which it was claimed that increased elephant densities at Addo did not in fact lead to tourism increases. However, this conclusion was based on a simple comparison of the strength of annual trends in tourists versus elephant densities, and did not (as we did) control for possible impacts of external driver variables.

Supplementary Note 3

Anecdotal evidence on recent African elephant declines and their negative impact on tourism.

As we have noted, data on tourist visits and their economic impacts at protected areas in Africa is sparse, even for the 1998-2007 period we used. Given how recent the phenomenon is, there does not appear to be any quantitative data which can help assess how far current elephant poaching is directly impacting tourist visits at individual protected areas. Nevertheless, anecdotal evidence suggests that the massive numbers of elephants lost over the last few years are already taking their toll on tourism, although it is difficult to separate the decline in attractiveness of protected areas due to fewer elephants versus that due to poaching activity itself. Private tourism operators have noted that major elephant poaching events at individual protected areas have led to a sharp reduction in tourist visits [7] and have also expressed concerns that current poaching levels may have devastating effects on tourism if not halted [8]. Elephant range country governments have also become concerned that the current poaching epidemic may threaten the tourism industry and the employment it generates [9,10].

Supplementary Note 4

Formulation of the Bayesian regression model of tourist visits to protected areas in Africa.

Following simplified notation for the exposition of Bayesian models [12], we model (natural-log-transformed) tourism visits y_i as a normally-distributed random variable, with mean u_i defined by a linear component including intercept α, elephant density ($E_{est,i}$), forest (F_i), and their interaction ($E_{est,i}F_i$), with respective coefficients β_E, β_F, and $\beta_{E\text{-}F}$, and a matrix of additional predictors $\mathbf{X}$ with vector of regression coefficients $\boldsymbol{\beta}$. Elephant density $E_{est,i}$ is modelled as a random variable with normal distribution having the observed population mean $E_{obs,i}$, and with standard deviation $E_{sd,i}$ where observed, and where missing modelled as a linear function of $E_{obs,i}$. Priors on intercepts and regression coefficients are noninformative normal distributions, and are noninformative Cauchy-distributed for variances (σ, σ_{sd}) [13].

$$y_i \sim \text{Normal}\,(u_i, \sigma) \tag{1}$$

$$u_i = \alpha + \boldsymbol{\beta}\mathbf{X_i} + \beta_E E_{est,i} + \beta_F F_i + \beta_{E\text{-}F} E_{est,i} F_i \tag{2}$$

$$E_{est,i} \sim \text{Normal}\,(E_{obs,i}, E_{sd,i}) \tag{3}$$

$$E_{sd,i} \begin{cases} = E_{sd,i} & \text{if } E_{sd,i} \text{ is estimated for population } i \\ \sim \text{Normal}\,(\alpha_{sd} + \beta_{sd} E_{obs,}, \sigma_{sd}) & \text{if } E_{sd,i} \text{ is missing} \end{cases} \tag{4}$$

$$\alpha \sim \text{Normal}\,(0,10) \tag{5}$$

$$\alpha_{sd} \sim \text{Normal}\,(0,10) \tag{6}$$

$$\boldsymbol{\beta} \sim \text{Normal}\,(0,10) \tag{7}$$

$$\beta_E \sim \text{Normal}\,(0,10) \tag{8}$$

$$\beta_F \sim \text{Normal}\,(0,10) \tag{9}$$

$$\beta_{E\text{-}F} \sim \text{Normal}\,(0,10) \tag{10}$$

$$\sigma \sim \text{Cauchy}\,(0,1) \tag{11}$$

$$\sigma_{sd} \sim \text{Cauchy}\,(0,1) \tag{12}$$

Supplementary References

1. Wittemyer G, Northrup JM, Blanc J, Douglas-Hamilton I, Omondi P, Burnham KP. Illegal killing for ivory drives global decline in African elephants. *PNAS* **111**, 13117-13121 (2014).

2. Stiles D, Martin R, Ji W, Moyle B. Analysis of ivory demand drivers. http://danstiles.org/publications/ivory/43.Ivory%20Analysis%20Final%20Report%20lo-res%20copy.pdf (2015).

3. Stiles D. The ivory trade and elephant conservation. *Environ. Conserv.* **31**, 309-321 (2004).

4. Vigne L, Martin E. *China faces a conservation challenge: the expanding elephant and mammoth ivory trade in Beijing and Shanghai*. Save the Elephants (2014).

5. Gabriel G, Hua N, Wang J. *Making a killing: A 2011 survey of ivory markets*. International Fund for Animal Welfare (2012).

6. Maciejewski K, Kerley GIH. Elevated elephant density does not improve ecotourism opportunities: convergence in social and ecological objectives. *Ecol. Appl.* **24**, 920-926 (2014).

7. Gettleman, J. *Elephants Dying in Epic Frenzy as Ivory Fuels Wars and Profits*. New York Times, Sept. 3 (2012).

8. Hammer, J. *The Fight Against Elephant Poachers Is Going Commando*. Smithsonian Magazine, June (2016).

9. Anonymous. *Mozambique: More Than 60 Poachers Arrested in Limpopo Park Last Year*. http://allafrica.com/stories/201605290265.html, May 29 (2016).

10. WildAid. *Tanzania: Poaching Threatens Tourism Industry Growth*. http://wildaid.org/news/tanzania-poaching-threatens-tourism-economy-growth, Aug. 11 (2015).

11. Anonymous. *Rhino poaching threatens tourism, economy*. http://www.fin24.com/Companies/TravelAndLeisure/Rhino-poaching-threatens-tourism-economy-20130920, Sept. 22 (2013).

12. McElreath R. Statistical rethinking: A Bayesian course with examples in R and Stan. Chapman and Hall/CRC (2015).

13. Stan Development Team. Stan modeling language: user's guide and reference manual. Version 2.9.0 (2015).
